# γ-Cyclodextrin/Genistein Inclusion Complex Catalyzes GPx4-Mediated Reduction of Organic/Inorganic Peroxides: Based on SERS and In Silico Research

**DOI:** 10.3390/foods15020297

**Published:** 2026-01-14

**Authors:** Mengmeng Zhang, Wenshuo Ren, Jingbo Liu, Yu Gao, Meng-Lei Xu, Ting Zhang

**Affiliations:** 1 Jilin Provincial Key Laboratory of Nutrition and Functional Food, College of Food Science and Engineering, Jilin University, Changchun 130062, China; mengmeng121388@163.com (M.Z.); renws24@mails.jlu.edu.cn (W.R.); ljb168@sohu.com (J.L.); 2 College of Plant Protection, Jilin Agricultural University, Changchun 130118, China; gaothrips@jlau.edu.cn; 3 State Key Laboratory of Supramolecular Structure and Materials, College of Chemistry, Jilin University, Changchun 130012, China

**Keywords:** genistein, γ-cyclodextrin, GPx4, degradation, inorganic peroxides, organic peroxides

## Abstract

Organic and inorganic peroxides can induce intracellular redox homeostasis. In this study, a γ-cyclodextrin/genistein inclusion complex (γ-CD/GEN) was constructed to systematically elucidate the molecular mechanism by which it catalyzes GPx4-mediated peroxide reduction. The results indicate that the incorporation of γ-CD effectively disrupts the aggregated state of GEN, achieving an encapsulation efficiency (EE) exceeding 40%. Surface-enhanced Raman spectroscopy (SERS) analysis reveals significant differences in the catalytic behavior of γ-CD/GEN toward cumene hydroperoxide (CHP) and hydrogen peroxide (H_2_O_2_): the reduction efficiency of CHP depends on both the concentration of γ-CD/GEN and GPx4, whereas the reduction of H_2_O_2_ is primarily regulated by the concentration of γ-CD/GEN. Isotope effect studies demonstrate that the reduction of CHP relies more on radical-initiated reactions, while the reduction of H_2_O_2_ involves proton transfer, with the differences in reduction rates correlating with their respective redox mechanisms. Molecular docking and molecular dynamics simulations further confirm that γ-CD/GEN can stably bind to the Sec (Cys)-46 site in the active center of GPx4, thereby enhancing its catalytic activity. This study provides a theoretical basis for the development of antioxidant strategies based on the precise regulation of enzyme activity.

## 1. Introduction

Organic peroxides (cumene hydroperoxide, CHP) and inorganic peroxides (H_2_O_2_) can generate reactive oxygen species (ROS) in living organisms, which may arise from metabolic processes or external stimuli. Excessive peroxides can disrupt cellular structures, alter enzyme activity, and trigger oxidative stress, thereby contributing to the development of various chronic diseases such as neurodegenerative disorders and cardiovascular diseases [1]. Therefore, developing highly effective antioxidants, particularly those targeting the reduction mechanism of peroxides, is of paramount importance.

Glutathione peroxidase 4 (GPx4) is a key selenoenzyme within the GPx family [2]. Its primary function is to catalyze the reduction of lipid peroxides via its active-site selenocysteine (Sec), playing a central role in maintaining cellular redox balance and mitochondrial function [3,4]. Concurrently, mitochondria serve as the primary site for reactive ROS production. The unsaturated fatty acids enriched in mitochondrial membranes are highly susceptible to peroxidation, and GPx4 can specifically scavenge mitochondrial membrane lipid peroxides, thereby maintaining mitochondrial membrane potential stability and normal physiological function [5,6]. Furthermore, GPx4 catalytic activity is regulated by multiple mechanisms: on one hand, its activity depends on the redox cycle of Sec and the continuous supply of glutathione (GSH). On the other hand, its activity can be upregulated via transcription factors such as Nrf2 [7,8]. Natural isoflavones such as genistein (GEN) have been shown to exert antioxidant effects by upregulating the expression and activity of GPx4 [9,10]. However, the molecular mechanism by which it directly interacts with the structure of GPx4 to modulate the conformation and catalytic efficiency of its active site has yet to be fully elucidated.

Surface-enhanced Raman scattering (SERS) is a rapid, non-destructive, and highly sensitive vibrational spectroscopy technique. It can precisely detect the unique vibrational signals of molecules and respond to subtle changes in the surrounding chemical environment [11,12]. SERS is capable of sensitively capturing the molecular structural and conformational information of proteins, small molecules, and their interactions. Moreover, it enables the monitoring of dynamic reaction processes under near-physiological conditions without interference from water molecules [13,14]. This provides an irreplaceable research tool for elucidating their interaction mechanisms at the molecular level. Similarly, Cui et al. employed SERS to investigate the ability of yellow mealworm protein to reduce organic/inorganic peroxides [15].

Based on this, the present study focuses on the core mechanism by which the γ-cyclodextrin/genistein inclusion complex (γ-CD/GEN) activates GPx4 and mediates the reduction of peroxides. First, the microstructure and encapsulation efficiency of γ-CD/GEN were characterized to clarify how γ-CD enhances GEN solubility. Subsequently, SERS was used to evaluate the effect of substrate concentration on peroxide reduction rates, and isotope effect analysis was employed to reveal the differences in the reduction mechanisms between CHP and H_2_O_2_. Cellular experiments further confirmed that γ-CD/GEN effectively enhances GPx enzyme activity in cells and mitochondria subjected to damage induced by different peroxides. Finally, molecular docking and molecular dynamics simulations were performed to elucidate the binding mode between γ-CD/GEN and GPx4, which corroborated the experimental findings. This study aims to provide a theoretical basis for the catalytic activation of GPx4 by natural bioactive compounds and to offer new insights into the treatment of oxidative stress-related diseases.

## 2. Materials and Methods

### 2.1. Materials and Chemicals

GEN (≥98% (*w*/*w*) purity) was obtained from Shanghai Yuanye Biotechnology Co., Ltd. (Shanghai, China). Recombinant GPx4 protein (human, purity (greater than 90% as determined by reducing SDS-PAGE)) was obtained from Cloud-Clone Crop (Wuhan, China). γ-CD, H_2_O_2_ (≥300 g/L of highly pure hydrogen peroxide dissolved in highly pure water), CHP (technical grade, ≥80% purity), sodium citrate (Na_3_C_6_H_5_O_7_, 99.8%), dimethyl sulfoxide (DMSO), silver nitrate (AgNO_3_, 99.0%) and deuterium oxide (D_2_O, 99.9%) were obtained from Sigma-Aldrich (Shanghai, China). Dulbecco’s modified eagle medium (DMEM), fetal calf serum (FBS), penicillin–streptomycin solution (P/S, the solution contains 10,000 units/mL penicillin and 10,000 μg/mL streptomycin) and phosphate-buffered saline (PBS) were obtained from Gibco (Grand Island, NY, USA). Total glutathione peroxidase assay kit with nicotinamide adenine dinucleotide phosphate (NADPH method, S0056) and cell mitochondrial isolation kit (C3601) were purchased from the Beyotime Biotechnology Co., Ltd. (Shanghai, China).

### 2.2. Apparatus

SERS were obtained using a confocal Raman spectrometer (LabRam ARAMIS, Yvon/HORIBA, Paris, France) with 633 nm excitation from a HeNe laser. The laser beam (1.0 mW power at the sample) was focused using a microscope with a 50× objective lens. The silicon wafer (band at 520.7 cm^−1)^ was used to calibrate the spectrometer, and the measurement range was from 500 to 1800 cm^−1^. The SERS was acquired using the Ag nanoparticles (Ag NPs) as described.

### 2.3. Synthesis of Ag NPs

Ag NPs were prepared according to the Lee and Meisel method [16]. Briefly, 36.00 mg of AgNO_3_ was added to 200 mL of distilled water and stirred manually. A total of 4 mL of sodium citrate solution (10 g/L) was added once boiling commenced. After heating for 40 min at 85 °C, a gray-green colloid mixture was formed, and the solution was cooled at room temperature (24 °C).

### 2.4. Preparation and Characterization of γ-CD/GEN

First, GEN solutions (20 and 40 mg/mL, respectively) were prepared in 75% ethanol, and an ultrasonic cell crusher (SCIENTZ-IID, Hangzhou, China) was used for sonication at 855 W for 10 s to ensure complete dissolution at room temperature (24 °C). Then, an aqueous solution of γ-CD (20 mg/mL) was prepared with distilled water. Under continuous manual stirring for 30 min, the GEN solution was added dropwise to the γ-CD solution (1:1 *v*/*v*, 24 °C) to give final concentrations of GEN in the γ-CD/GEN solution of 10 and 20 mg/mL, respectively, in order to obtain self-assembled γ-CD/GEN inclusion complexes [9]. The final concentrations in the spectral analysis were made to be 100 and 200 μg/mL. GEN, γ-CD and γ-CD/GEN were morphologically evaluated using transmission electron microscopy (TEM, HT 7820 Hitachi, Tokyo, Japan). Typically, the 10 μL sample is slowly pipetted onto the surface of the carbon film on the copper grid. The sample is then left to stand at room temperature (24 °C) for 30 min to allow the sample to adsorb fully onto the carbon film. Observation is subsequently performed (TEM voltage: 120 kV; chamber pressure: 10^−5^–10^−7^ Pa).

### 2.5. Encapsulation Efficiency (EE) Assay

To further characterize the EE of GEN in γ-CD/GEN at concentrations in the range of 0.4–1.6 mg/mL, the EE of GEN was quantitatively determined by establishing a standard curve (Y = 0.1312x + 0.0172, R^2^ > 0.99) with a UV–Vis spectrophotometer (UV-2250, Shimadzu, Japan) at 260 nm. Specifically, freshly prepared γ-CD/GEN was centrifuged (10,000× *g*, 5 min, 24 °C) to remove precipitated GEN and insoluble aggregates. The supernatant was then extracted for GEN content determination [17]. The EE of γ-CD/GEN was calculated by the following equation:

(1) EE (%) = entrapped GEN/total self-assembly × 100%

### 2.6. Measurement of γ-CD/GEN-Catalyzed GPx4-Mediated Reduction of Peroxides

According to the method of Orian et al., polyphenols were determined to catalyze the GPx4-mediated reduction of peroxides with slight modifications [18]. Specifically, activity assays were performed in H_2_O and D_2_O phases, respectively. All reagents in the Total Glutathione Peroxidase Assay Kit were dissolved in D_2_O to maintain the D_2_O phase. Different concentrations of H_2_O_2_ (range 3–6 mM), CHP (range 3–6 mM), GPx4 (range 10–20 μg/mL) and γ-CD/GEN (range 0.75–1.5 mM) were used for treatment. Subsequently, glutathione peroxidase buffer, NADPH, GSH and glutathione reductase (GR) were added to the samples according to the instructions of the total glutathione peroxidase assay kit, along with the same volume of Ag NPs (60 nm) [19]. SERS of the samples were acquired using a He–Ne laser (633 nm, laser power 50 mW, exposure time 10 s, 10 cumulative scans).

### 2.7. Cell Culture and Treatment

PC12 (derived from rat adrenal medullary tumor cells, highly differentiated) cells were obtained from the national collection of authenticated cell cultures and cultured in DMEM containing 10% FBS and 1% P/S at 37 °C in a medium containing 95% air and 5% CO_2_. PC12 cells were pre-cultured for 12 h. Subsequently, the cells were treated with different concentrations of γ-CD/GEN (0.4 and 1.6 mg/mL) for 12 h. Finally, the cells were exposed to 14 mM H_2_O_2_ and 280 μM CHP according to a previous study to bring the cell viability close to 50% [9]. Control cells were treated with an equal volume of DMEM.

### 2.8. Measurement of Intracellular GPx Enzyme Activity

PC12 cells (1 × 10^6^ cells/well) were seeded into 6-well plates at 1600 μL per well and allowed to adhere for 12 h (at 37 °C in an incubator containing 95% air and 5% CO_2_). Firstly, cells were treated with 200 μL of 0.4 or 1.6 mg/mL of γ-CD/GEN for 12 h to give final concentrations of 100 and 400 μg/mL in the cells, expressed as γ-CD/GEN-L and γ-CD/GEN-H, respectively, followed by treatment with 200 μL of CHP (280 μM) or H_2_O_2_ (14 mM) for 12 h, resulting in a final concentration of CHP (28 μM) or H_2_O_2_ (1.4 mM). CHP and H_2_O_2_ were both diluted in DMEM containing 10% FBS and 1% P/S. GPx activity in the cells was measured according to the instructions of the total glutathione peroxidase assay kit (NADPH method). For the measurement of GPx activity in mitochondria, mitochondria were first isolated according to the instructions of the cell mitochondrial isolation kit to obtain high-purity mitochondria, followed by measurement of GPx activity within these mitochondria. The intramitochondrial GPx activity was measured using the same method as the intracellular GPx activity.

### 2.9. Molecular Docking of γ-CD/GEN with GPx4

The structure file for the GPx4 protein (PDB ID: 2OBI) is available from the PDB protein database, while the γ-CD (PubChem CID: 5287407) molecule and the GEN (PubChem CID: 5280961) molecule are available from the Pubchem website. Use PyMOL (3.0) to pre-process protein structures, removing impurities (solvents, ions, small molecules, etc.) from the protein and repairing the protein structure to obtain the protein ready for docking. Receptor (GPx4) and ligand (GEN and γ-CD/GEN) structures for molecular docking were prepared using Autodock Tools-1.5.7 [20], with the PDBQT-formatted receptor and ligand files serving as input for the molecular docking. Docking was simulated by using the AutoDock Vina 1.2.5 program [21], with rigid receptor and flexible ligand models. The box dimensions were 6.4 nm × 6.3 nm × 6.8 nm, grid steps were 0.375 nm, and the center location was X = 15.4 Å, Y = 28.8 Å, Z = 42.0 Å. After docking, the conformation corresponding to the optimal molecular docking score was selected as the best conformation for analysis.

### 2.10. Molecular Dynamics (MD) Simulations

MD simulations were performed using Gromacs 2021 software [22]. The force field used for the system was AMBER99sb-ildn, while the γ-CD and GEN molecules used the GAFF force field. The system molecules (8732 H_2_O molecules and one Cl ion) were placed within a box using GROMACS (6.4 nm × 6.3 nm × 6.8 nm). The MD simulations were performed at 298.15 K temperature and 1 bar pressure. Energy minimisation, typical ensemble (NVT, 100 ps) and isothermal isobaric (NPT, 100 ps) pre-equilibria were performed sequentially prior to the simulation. All systems were simulated for 100 ns of production simulation. The production simulation used the leap-frog algorithm to integrate the Newtonian equations of motion, with a time step of 2 fs. The V-rescale method was applied for temperature coupling, and the Parrinello–Rahman method was used for pressure coupling. The neighbor search was performed using the Verlet method, with Coulombic and van der Waals interaction cut-off radii set to 1.2 nm. Long-range electrostatic interactions were treated using the PME method. Long-range dispersion corrections were applied to energy and pressure. After the MD simulation, the trajectories were analyzed using GROMACS 2021 software to calculate the root mean square difference (RMSD), root mean square fluctuation (RMSF), solvent-accessible surface area (SASA), hydrogen bonding (H-Bond), interaction energy and binding free energy (MM-PBSA).

### 2.11. Statistical Analyses

At least three independent experiments were performed (*n* ≥ 3). Results are shown as mean ± standard deviation. ANOVA, or unpaired Student’s *t*-test, was used to indicate the differences between means. Statistical significance was set at *p* < 0.05.

## 3. Results and Discussion

### 3.1. Microscopic Structure and EE of γ-CD/GEN

**Figure 1:**
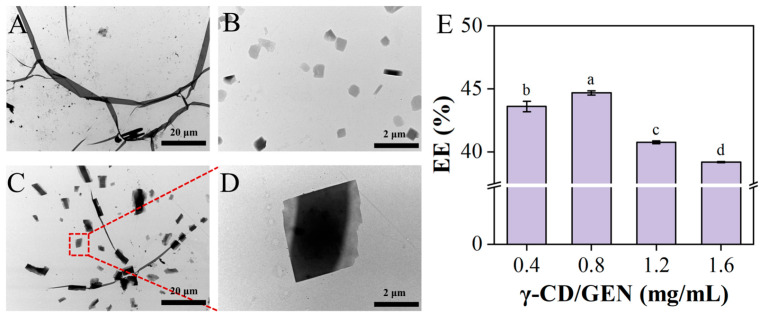
Microscopic morphology of GEN, γ-CD, and γ-CD/GEN, and EE of γ-CD/GEN. (**A**) TEM images of GEN; (**B**) TEM images of γ-CD and (**C**,**D**) TEM images of γ-CD/GEN; (**E**) EE of γ-CD/GEN. Different letters indicate significant differences. All data represent the mean ± SD of three independent experiments. Key: different letters indicate the significance between groups.

To investigate whether the introduction of γ-CD can effectively break the aggregated state of GEN, TEM observations were performed on GEN, γ-CD, and γ-CD/GEN, with the results shown in Figure 1. Figure 1A shows that GEN exhibits a fibrous structure at 700× and forms aggregates. In Figure 1B, the γ-CD image appears as uniformly dispersed particles. Figure 1C,D shows the microstructure of γ-CD/GEN. The addition of γ-CD causes GEN to disperse into smaller particles due to intermolecular forces (H-bonds and hydrophobic interactions) between GEN and γ-CD, allowing GEN to enter the interior of the γ-CD cavities [23]. Furthermore, the results in Figure 1E show that the EE of the γ-CD/GEN inclusion complex is greater than 40% at different concentrations. Current research has found that GEN is a suitable guest molecule for cyclodextrin, with its solubility significantly enhanced after encapsulation [24,25,26]. In summary, the inclusion effect of γ-CD with GEN disrupts the aggregation of GEN and enhances its solubility, which provides a potential basis for the interaction between γ-CD/GEN and GPx4.

### 3.2. Study on the Effect of Substrate Concentration on Reduction Rate

#### 3.2.1. H_2_O Phase

**Figure 2:**
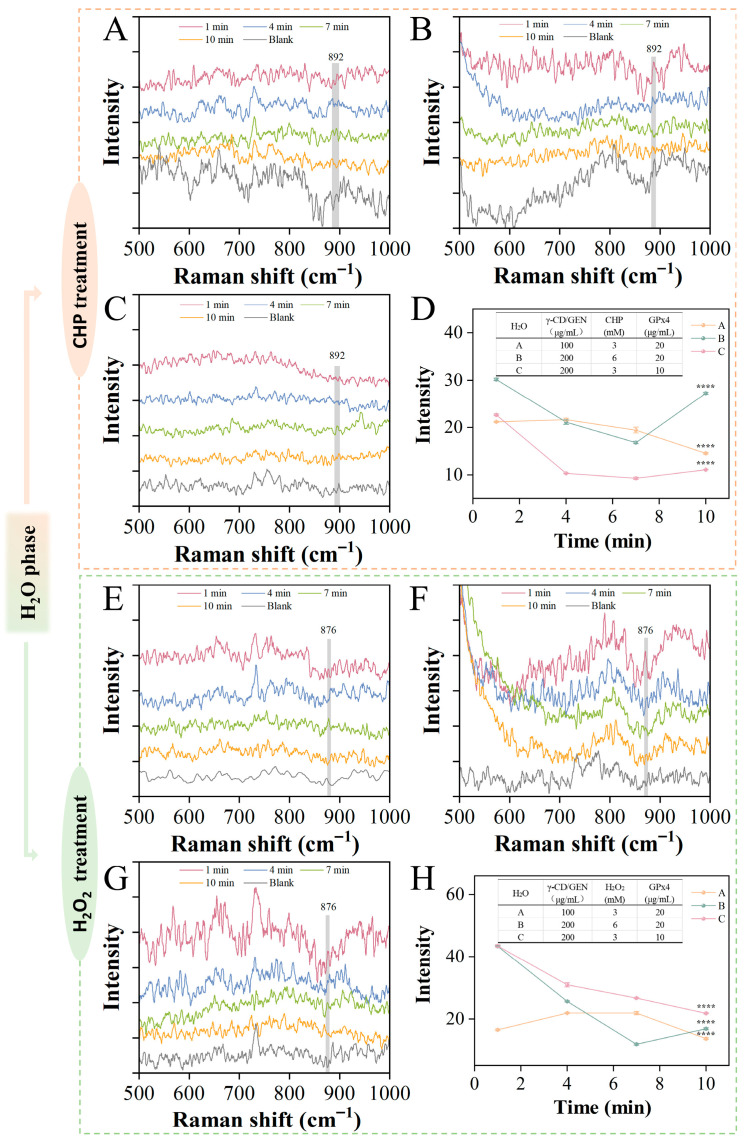
SERS (500–1800 cm^−1^) of GPx4-mediated organic/inorganic reduction of peroxide by γ-CD/GEN in an H_2_O phase within 10 min of the reaction. (**A**) Results for 100 μg/mL γ-CD/GEN + 3 mM CHP + 20 μg/mL GPx4; (**B**) 200 μg/mL γ-CD/GEN + 6 mM CHP + 20 μg/mL GPx4; (**C**) 200 μg/mL γ-CD/GEN + 3 mM CHP + 10 μg/mL GPx4 and (**D**) SERS bond strength at 892 cm^−1^; (**E**) 100 μg/mL γ-CD/GEN + 3 mM H_2_O_2_ + 20 μg/mL GPx4; (**F**) 200 μg/mL γ-CD/GEN + 6 mM H_2_O_2_ + 20 μg/mL GPx4; (**G**) 200 μg/mL γ-CD/GEN + 3 mM H_2_O_2_ + 10 μg/mL GPx4; and (**H**) 876 cm^−1^ at the SERS bond strength. All data represent the mean ± SD of three independent experiments. Key: **** *p* < 0.0001 indicates significant difference compared to the same group at 1 min.

SERS were used to analyze the reaction mixtures. Ag NPs can enhance the peak intensity through localized surface plasmon resonance, while no significant signal from the silver nanoparticles was observed at 876 cm^−1^ and 892 cm^−1^ (Figure S1). Figure 2 shows the effect of different substrate concentrations on the reduction rate. The Raman intensity at 892 cm^−1^ or 876 cm^−1^ was monitored during the interaction of CHP (ranging from 3 to 6 mM), H_2_O_2_ (range 3 to 6 mM), GPx4 (range 10 to 20 μg/mL), and γ-CD/GEN (range 0.75–1.5 mM) in an H_2_O phase [27]. Typically, the characteristic peak in the 860–890 cm^−1^ range is related to the *v*O-O molecular vibration mode of peroxides. The higher the intensity of the characteristic peak, the higher the concentration of peroxides, which is consistent with previous studies [28,29]. Figure 2A–C shows the Raman intensity variations in γ-CD/GEN, CHP and GPx4 reactions. Figure 2D shows the Raman intensity of the above mixture at 892 cm^−1^. By comparing line A and line C in Figure 2D, it was observed that within 10 min of the reaction, the Raman intensity of line A decreased by 31% (from 21.18 ± 0.12 to 14.54 ± 0.12), while line C decreased by 51% (from 22.66 ± 0.17 to 11.12 ± 0.04). In contrast, the Raman intensity of line B decreased by only 10% (from 30.17 ± 0.27 to 27.22 ± 0.22) and showed a rebound at the 7 min mark. We speculate that higher concentrations of γ-CD/GEN and GPx4 can synergistically promote the reduction of CHP, as γ-CD/GEN may bind to the active center of GPx4, thereby activating its catalytic activity. However, when the concentration of CHP is too high, the reaction system cannot fully reduce it. The incompletely reduced CHP may accumulate in the system and form intermediate products, leading to the rebound of the curve.

Figure 2E–G shows the Raman intensity of the reaction of γ-CD/GEN, H_2_O_2_ and GPx4, and Figure 2H shows the Raman intensity of the above mixture at 876 cm^−1^. By comparing line A with lines B and C in Figure 2H, it was observed that the Raman intensities of lines B and C decreased significantly in the early stage of the reaction, dropping by 61% (from 43.44 ± 0.18 to 16.83 ± 0.28) and 50% (from 43.52 ± 0.37 to 21.81 ± 0.20), respectively. In contrast, the Raman intensity of line A decreased by only 17% (from 16.55 ± 0.27 to 13.67 ± 0.26) within 10 min of the reaction. This indicates that a high concentration of γ-CD/GEN can rapidly reduce H_2_O_2_ and exhibits a greater extent of reduction in the initial phase of the reaction. For the H_2_O_2_ system, γ-CD/GEN may significantly enhance the catalytic rate at the early stage, possibly by influencing the proton-transfer process at the active center of GPx4 or directly participating in the reduction step. In summary, the reduction behaviors of the two peroxides differ. The reduction rate of CHP is co-regulated by the concentrations of γ-CD/GEN and GPx4, whereas the reduction rate of H_2_O_2_ is predominantly governed by the concentration of γ-CD/GEN, a trend that becomes especially evident under higher substrate concentrations.

#### 3.2.2. D_2_O Phase

**Figure 3:**
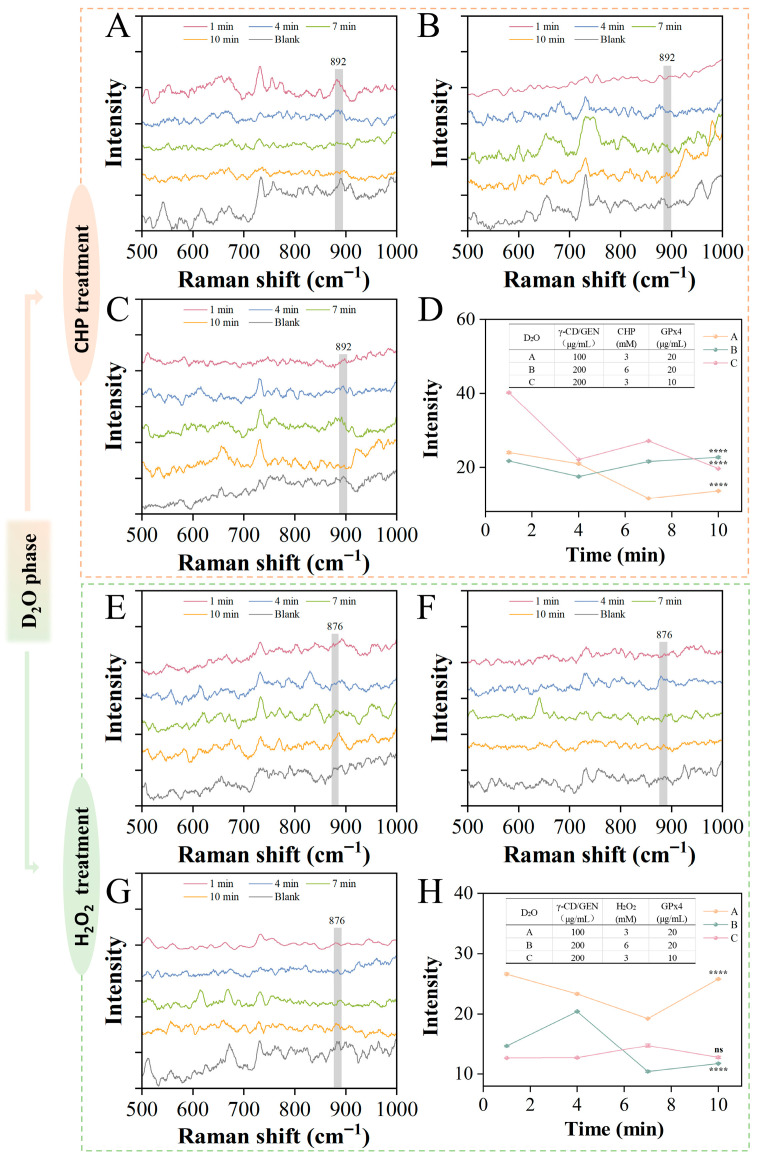
SERS (500–1800 cm^−1^) of GPx4-mediated organic/inorganic reduction of peroxide by γ-CD/GEN in a D_2_O phase within 10 min of the reaction. (**A**) The results for 100 μg/mL γ-CD/GEN + 3 mM CHP + 20 μg/mL GPx4; (**B**) 200 μg/mL γ-CD/GEN + 6 mM CHP + 20 μg/mL GPx4; (**C**) 200 μg/mL γ-CD/GEN + 3 mM CHP + 10 μg/mL GPx4 and (**D**) SERS bond strength at 892 cm^−1^; (**E**) 100 μg/mL γ-CD/GEN + 3 mM H_2_O_2_ + 20 μg/mL GPx4; (**F**) 200 μg/mL γ-CD/GEN + 6 mM H_2_O_2_ + 20 μ g/mL GPx4; (**G**) 200 μg/mL γ-CD/GEN + 3 mM H_2_O_2_ + 10 μg/mL GPx4; and (**H**) 876 cm^−1^ at the SERS bond strength. All data represent the mean ± SD of three independent experiments. Key: **** *p* < 0.0001 indicates significant difference compared to the same group at 1 min.

To investigate the differences between γ-CD/GEN and GPx4 in the reduction of two types of hydrogen peroxide, the reaction dynamics in the D_2_O phase were monitored in situ, and the isotope effect was used to conduct an in-depth study of the substrate reduction process and the changes in reduction rate. In D_2_O, D (an isotope of H) replaces H. Since D has a larger mass than H, the D-O bond has a lower vibrational frequency compared to the H-O bond, which may lead to a change in the rate of reaction [30]. Figure 3A–D show the Raman intensity of the interaction between CHP, GPx4, and γ-CD/GEN in the D_2_O phase. By comparing line A and line C in Figure 3D, it was found that the Raman intensity of the A decreased by 43% (from 23.97 ± 0.32 to 13.67 ± 0.15) within 10 min, while the Raman intensity of line C decreased by 51% (from 40.25 ± 0.14 to 19.64 ± 0.06). In comparison, the reduction rate in the D_2_O phase was higher than that in the H_2_O phase, likely due to interactions such as H-bonding between γ-CD/GEN and GPx4, which modified the activity of GPx4. The stronger H-bond in D_2_O may enhance the activity of GPx4, thereby increasing the degradation rate. In addition, line B in Figure 3D could not be fully reduced due to the high concentration of CHP, either in the H_2_O phase or the D_2_O phase. In Figure 3H, the Raman intensities of lines A, B, and C show little change at the reaction onset and after 10 min of reaction, decreasing by 3% (26.61 ± 0.17 to 35.78 ± 0.09), 20% (from 14.68 ± 0.13 to 11.77 ± 0.06), and 0% (from 12.67 ± 0.02 to 12.78 ± 0.17), respectively. This indicates that the reduction rate of H_2_O_2_ is relatively slow in the D_2_O phase, regardless of changes in substrate concentration. This is because the reduction of H_2_O_2_ results in the formation of water and oxygen, a process that typically involves proton (hydrogen atom) transfer. In D_2_O, the larger mass of deuterium (D) requires higher energy for the reaction, thereby slowing down the reduction rate. In contrast, the degradation of CHP primarily relies on radical-induced polymerization reactions rather than proton-transfer pathways, and these reactions are unaffected by the isotope effect [31]. As a result, the reduction rate of CHP may be faster in D_2_O compared to H_2_O. In summary, the differences in the reduction rates of CHP and H_2_O_2_ in different media are closely related to their redox mechanisms, which is somewhat explained in the D_2_O phase.

### 3.3. Study on the Effects of γ-CD/GEN on GPx Activity in Cells or Mitochondria

Multiple members of the GPx family (e.g., GPx1, GPx2, GPx3, GPx4, etc.) usually occur together in cells, and therefore, GPx activity measured in cells reflects the overall activity of these subtypes. Although different GPx subtypes have functional differences, all GPx subtypes rely on GSH to reduce peroxides, thereby protecting cells from oxidative damage. Among them, GPx4, as a unique subtype, exists in different cellular compartments such as the cytoplasm (cGPX4), mitochondria (mGPX4), and the nucleus (nGPX4), where it plays an important antioxidant role in each region. Especially in the mitochondrial compartment, mGPx4 prevents mitochondrial membrane damage by reducing the accumulation of lipid peroxides, thereby maintaining mitochondrial integrity [32]. Moreover, GPx1 and GPx4 are the predominant isoforms in cells, whilst GPx2 and GPx3 are present at lower levels. Within the mitochondria, GPx4 constitutes the absolutely dominant isoform; consequently, GPx activity in the mitochondria can also indirectly reflect that of GPx4.

**Figure 4:**
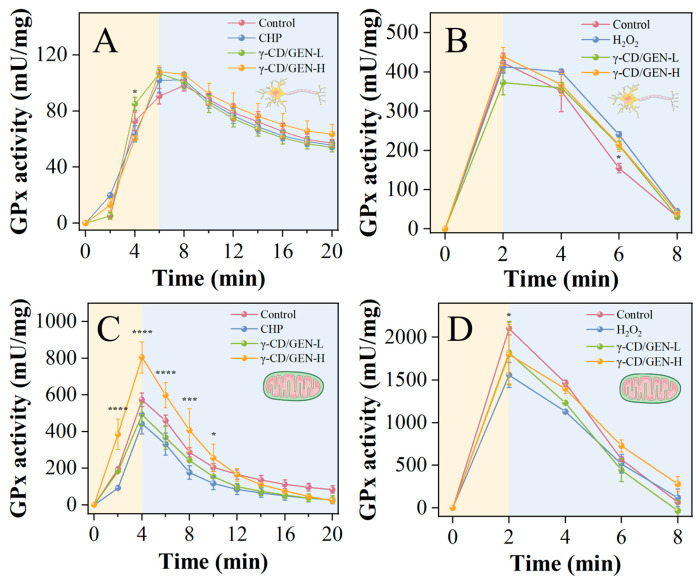
GPx enzyme activities in PC12 cells and purified mitochondria isolated from PC12 cells. (**A**) GPx activity in PC12 cells after CHP treatment; (**B**) GPx activity in PC12 cells after H_2_O_2_ treatment; (**C**) GPx4 activity in mitochondria after CHP treatment and (**D**) GPx4 activity in mitochondria after H_2_O_2_ treatment. All data represent the mean ± SD of three independent experiments. Key: * *p* < 0.05, *** *p* < 0.001, and **** *p* < 0.0001 indicate significant differences compared with the CHP/H_2_O_2_ group.

According to previously validated models, the inhibition rate of PC12 cells reaches 50% when the concentration of H_2_O_2_ is 14 mM or the concentration of CHP is 280 μM. Meanwhile, γ-CD/GEN exhibits cytotoxicity when the concentration exceeds 400 μg/mL [9]. Notably, our previous studies have confirmed that γ-CD/GEN possesses a distinct ability to upregulate GPx4 in cells [9]. To further explore its potential to enhance GPx enzyme activity within cells or mitochondria across both oxidative stress models, concentrations of 100 μg/mL (low dose) and 400 μg/mL (high dose) were selected for intervention. As shown in Figure 4A–D, after treating cells and mitochondria with different peroxides (CHP and H_2_O_2_), GPx activity was increased within a short period of time in different treatment groups. This suggests that peroxides, as sources of oxidative stress, can stimulate cells to cope with oxidative stress by increasing antioxidant enzyme activities. The decrease in GPx activity with prolonged treatment time reflects the gradual depletion of antioxidant enzymes [33]. Specifically, the enzyme activity of cells or mitochondria treated with CHP reaches its peak within 4 or 6 min, while the enzyme activity of H_2_O_2_-treated cells peaks within 2 min. Subsequently, the enzyme activity after CHP treatment gradually decreases and remains steady for 14 or 16 min, whereas the enzyme activity after H_2_O_2_ treatment rapidly declines within 6 min. This suggests that CHP maintains enzyme activity for a longer period through slow and sustained oxidation, while H_2_O_2_, as a strong oxidant, decomposes rapidly to generate hydroxyl radicals, leading to a sharp decline in enzyme activity within a short time. Furthermore, in both CHP-treated cells and mitochondria, the GPx activity in the γ-CD/GEN-H group was generally higher than that in the control and model groups (Figure 4A,C). However, in H_2_O_2_-treated cells, the GPx activity in the γ-CD/GEN-H group was lower than that in the model group (Figure 4B), whereas in H_2_O_2_-treated mitochondria, the GPx activity in the γ-CD/GEN-H group was higher than that in the model group (Figure 4D). This difference is related to the mechanisms by which γ-CD/GEN exerts its effects in different mitochondrial models. In CHP-induced injury, γ-CD/GEN-H may have promoted GPx activity through its scavenging or binding of lipid peroxides; whereas in H_2_O_2_-induced injury, H_2_O_2_ may have caused more direct and severe oxidative damage to mitochondria, thereby inhibiting GPx activity [34,35]. In summary, γ-CD/GEN exhibits a promoting effect on GPx enzyme activity following CHP- and H_2_O_2_-induced damage, which is closely related to the type of peroxides and the cellular environment. These findings provide a theoretical basis for the potential application of γ-CD/GEN in oxidative stress-related diseases.

### 3.4. Molecular Docking Reveals the Binding of γ-CD/GEN to the Active Site of GPx4

**Figure 5:**
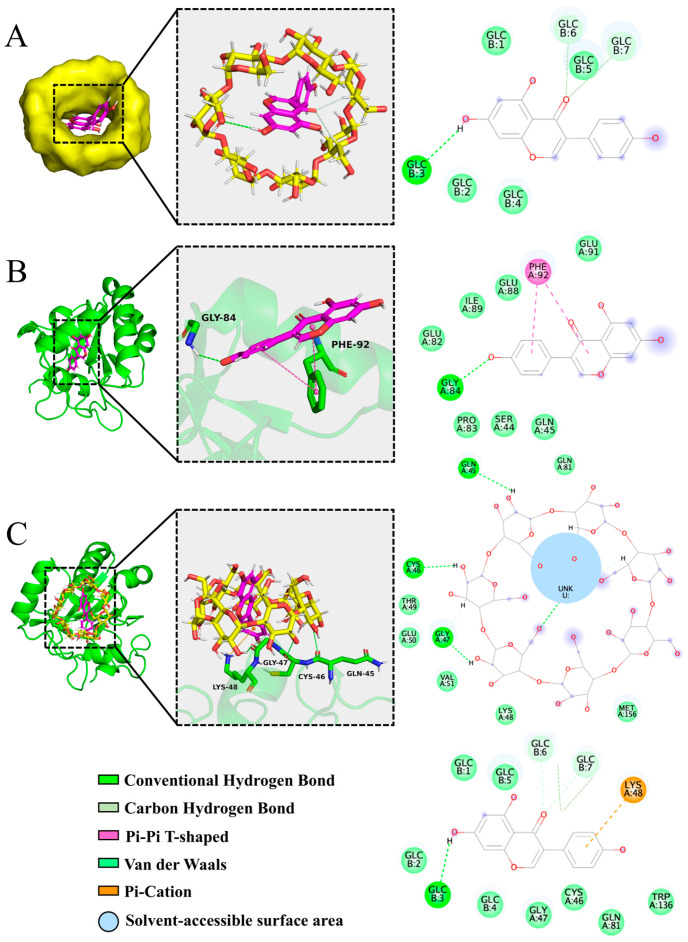
Molecular docking analysis. (**A**) The 3D and 2D interaction diagrams of γ-CD and GEN molecular docking, with yellow representing the γ-CD molecule and pink representing the GEN molecule; (**B**) 3D and 2D interaction diagrams of GPx4 protein and GEN molecular docking, with green representing GPx4; (**C**) 3D and 2D interaction diagrams of GPx4 protein and γ-CD/GEN molecular docking.

Molecular docking serves as a tool to aid in understanding intermolecular interactions and predicting molecular binding [36]. As a key antioxidant enzyme, GPx4 primarily facilitates the reduction of peroxides through catalysis at its active site. The core residues of its active site include Sec46, Gln-81, Trp-136, and Asn-137 [37]. However, in the GPx4 structure used here (PDB: 2OBI), the opal termination codon (UGA) that normally encodes selenocysteine (Sec) has been mutated to a triplet (UGU)-encoding cysteine. Consequently, the Sec at this position is replaced by a cysteine (Cys) residue in the expressed protein. Shown in Figure 5A, in the optimal binding mode, GEN binds to the hydrophobic cavity region of γ-CD via hydrogen bonding with a minimum binding energy of −6.045 kcal/mol, indicating a strong and stable interaction between γ-CD and GEN. Subsequently, both GPx4-GEN and GPx4-γ-CD/GEN complexes were docked, respectively, and are shown in Figure 5B,C. Specifically, the GEN molecule binds to Gly-84 of GPx4 via hydrogen bonding and also to Phe-92 via non-covalent interactions (π–π interactions) with a minimum binding energy of −5.827 kcal/mol. In contrast, the γ-CD/GEN inclusion complex binds to amino acid residues such as Lys-48, Gly-47, Sec (Cys)-46, and Gln-45 via hydrogen bonds, van der Waals forces, or π–cation interactions, exhibiting a minimum binding energy of −8.156 kcal/mol. This indicates a more stable binding between γ-CD/GEN and GPx4, which primarily benefits from the introduction of γ-CD that expands the interaction interface and forms a network of non-covalent bonds. More importantly, γ-CD/GEN binds directly to the active-site residue Sec (Cys)-46, suggesting that it can influence the structure and function of the catalytic center of GPx4. In summary, molecular docking reveals that, compared with GEN alone, γ-CD/GEN possesses stronger GPx4-binding affinity and more precise targeting of the active site, providing a theoretical basis for the experimentally observed enhancement of enzymatic activity.

### 3.5. Study of the Binding Mechanism of γ-CD/GEN to GPx4 Based on MD Simulation

**Figure 6:**
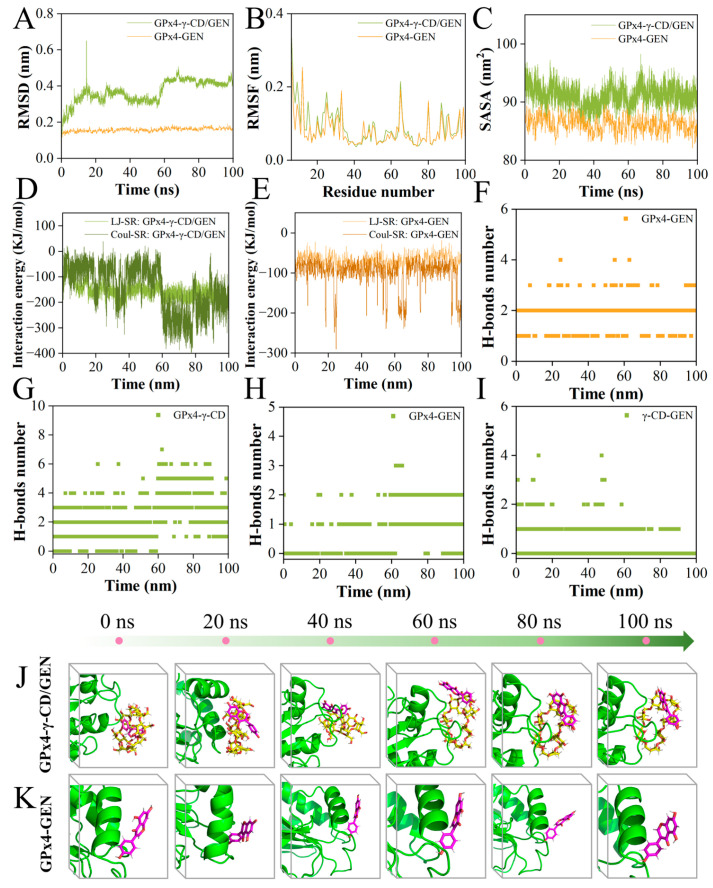
MD simulation analysis of GPx4 protein with GEN or γ-CD/GEN. RMSD curves (**A**), RMSF curves (**B**) and SASA curves (**C**) of GPx4-γ-CD/GEN and GPx4-GEN; interaction forces of GPx4-γ-CD/GEN (**D**) and GPx4-GEN; (**E**) hydrogen bonding changes in GPx4-GEN in GPx4 versus GEN simulations; (**F**) hydrogen bonding changes in GPx4-γ-CD (**G**), GPx4-GEN (**H**), and γ-CD-GEN (**I**) in GPx4 versus γ-CD/GEN simulations; snapshots of different simulation moments of GPx4-γ-CD/GEN (**J**) and GPx4-GEN (**K**).

Based on the results of molecular docking, 100 ns MD simulations were performed to investigate the dynamic properties of GPx4 proteins with GEN or γ-CD/GEN. The results of the MD simulations are essential for assessing factors such as the structural stability of GPx4 proteins upon binding to small molecules and the hydrophobicity of amino acid residues [38]. Among them, RMSD shows the conformational changes between GPx4 and GEN or γ-CD/GEN during the simulation. The trend of change is an important feature to determine whether the simulation has reached stability. Figure 6A shows that the RMSD curves of GPx4 with GEN molecules were stable at 0.1–0.2 nm throughout the process, while GPx4 with γ-CD/GEN remained stable at 60–100 ns. This process was supported by conformational maps at different moments (Figure 6J,K). RMSF is an indicator to evaluate the flexibility of protein amino acids throughout the simulation process, and larger RMSF values indicate the greater flexibility of amino acid residues [39]. As shown in Figure 6B, the RMSF of GPx4 with γ-CD/GEN was slightly larger compared to that of GPx4 with GEN, due to the introduction of γ-CD changing the contact surface of GPx4 with GEN. These changes may have led to the involvement of more amino acid residues, which increased the flexibility of these regions and ultimately manifested as a larger RMSF, which is consistent with the molecular docking results (Figure 5B,C). Solvent-accessible surface area (SASA) quantifies the surface area of a biomolecule accessible to the solvent, serving as an indicator of the degree of desolvation during co-assembly [40]. SASA values of GPx4-γ-CD/GEN were higher than those of GPx4-GEN, as shown in Figure 6C, confirming the hydrophilic nature of γ-CD. Shown in Figure 6D,E, the interaction energy consists of van der Waals interactions (LJ-SR) and Coulomb interactions (Coul-SR), with smaller values indicating stronger interactions. The LJ-SR energy of GPx4-γ-CD/GEN is −181.25 kJ/mol, and the Coulomb-SR energy is −183.22 kJ/mol. The average LJ-SR is −158.65 kJ/mol, and the average Coulomb-SR is −145.11 kJ/mol. In contrast, the LJ-SR energy of GPx4-GEN is −74.81 kJ/mol, and the Coulomb-SR energy is −192.51 kJ/mol. The average LJ-SR is −69.30 kJ/mol, and the average Coulomb-SR is −100.74 kJ/mol. Furthermore, the binding free energy of GPx4-γ-CD/GEN is −35.27 kcal/mol, while that of GPx4-GEN is −22.97 kcal/mol (Table S1). These results suggest that GPx4-γ-CD/GEN exhibits stronger and more balanced interactions with higher stability, whereas GPx4-GEN is mainly governed by electrostatic interactions and has lower stability. H-bonds between protein receptors and small molecule ligands contribute to the stability of the complex. In the GPx4-GEN MD simulation, the maximum number of H-bonds was 4 (Figure 6F), whereas in the GPx4-γ-CD/GEN MD simulation, the maximum number of H-bonds was 7 (Figure 6G–I), suggesting that the higher the number of H-bonds, the more stable it is. In conclusion, the introduction of γ-CD enhances the binding stability between GPx4 and γ-CD/GEN, increases the flexibility of amino acid residues near the binding interface, demonstrates the hydrophilic properties of γ-CD, and increases the number of hydrogen bonds formed. This contributes to improving the overall stability of the GPx4-γ-CD/GEN complex. These conclusions support the findings from molecular docking analysis.

## 4. Conclusions

This study systematically investigates the interaction between the γ-CD/GEN and GPx4, focusing on their collaborative catalysis of the reduction of organic/inorganic peroxides. Overall, γ-CD effectively disrupts the aggregation of GEN, thereby facilitating the self-assembly of the γ-CD/GEN inclusion complex, with an EE exceeding 40%. Furthermore, SERS revealed that the interaction between γ-CD/GEN and GPx4 efficiently reduces both peroxides within 10 min. In the H_2_O phase, the concentrations of γ-CD/GEN and GPx4 were found to determine the reduction rate of CHP, while the concentration of γ-CD/GEN alone governed the reduction rate of H_2_O_2_. In the D_2_O phase, the reduction rates of both CHP and H_2_O_2_ were revealed to correlate with their respective redox mechanisms: CHP reduction relied more heavily on radical reactions, whereas H_2_O_2_ reduction depended on proton transfer. Cellular experiments confirmed that γ-CD/GEN effectively enhances GPx enzyme activity in both cells or mitochondria induced by CHP, but only improves GPx enzyme activity in mitochondria following H_2_O_2_ exposure. Computational simulations further demonstrated that the -OH groups in γ-CD/GEN form covalent interactions that stabilize the binding to the active site Sec (Cys)-46 of GPx4, thereby enhancing its catalytic activity. Overall, γ-CD/GEN plays a key role in catalyzing the reduction of GPx4-mediated peroxides.

## Data Availability

The original contributions presented in the study are included in the article and Supplementary Materials; further inquiries can be directed to the corresponding authors.

## Supplementary Materials

The following supporting information can be downloaded at: https://www.mdpi.com/article/10.3390/foods15020297/s1, Figure S1: SERS of AgNPs. Table S1: Free energy of binding of GPx4 to two ligands (kcal/mol).
