# Supplementary material for: γ-Cyclodextrin/Genistein Inclusion Complex Catalyzes GPx4-Mediated Reduction of Organic/Inorganic Peroxides: Based on SERS and In Silico Research
Source: Foods. 2026 Jan 14;15(2):297. doi: 10.3390/foods15020297 (PMC12840367; doi:10.3390/foods15020297)
Supplement: Supplementary file 1 [file foods-15-00297-s001.zip › foods-3971745-supplementary.pdf]

Supplementary Information

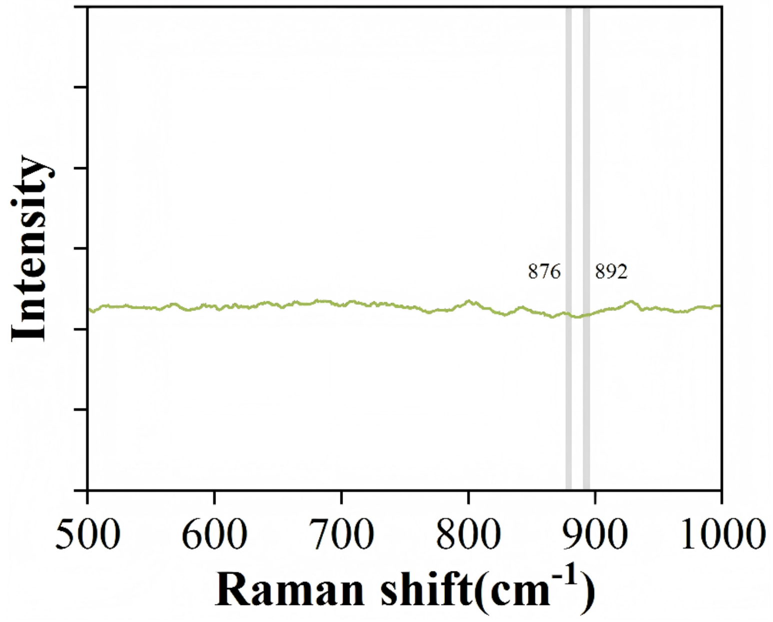

Figure S1. SERS spectroscopy of AgNPs.

Table S1. Free energy of binding of GPx4 to two ligands (kcal/mol)

Energy component	GPx4-γ-CD/GEN	GPx4-GEN
ΔVDWAALS	-45.51	-16.25
ΔEEL	-69.06	-26.89
ΔEGB	84.71	23
ΔESURF	-5.4	-2.84
ΔGGAS	-114.58	-43.13
ΔGSOLV	79.31	20.16
ΔTOTAL	-35.27	-22.97

ΔVDWAALS, van der Waals energy; ΔEEL, electrostatic energy; ΔEGB, polar solvation energy; ΔESURF, nonpolar solvation energy; ΔGGAS, gas molecular energy = ΔVDWAALS + ΔEEL; ΔGSOLV, energy of solvation = ΔEGB + ΔESURF; ΔTOTAL, total binding free energy = ΔGGAS + ΔGSOLV.
